# Foot-and-mouth disease: overview of motives of disease spread and efficacy of available vaccines

**DOI:** 10.1186/s40781-015-0042-8

**Published:** 2015-04-01

**Authors:** Ali Saeed, Sehrish Kanwal, Memoona Arshad, Muhammad Ali, Rehan Sadiq Shaikh, Muhammad Abubakar

**Affiliations:** Instituteof Molecular Biology and Biotechnology, Bahauddin Zakariya, University, Multan, Pakistan; National Institute for Biotechnology and Genetic Engineering, (NIBGE), Faisalabad, Pakistan; National Veterinary Laboratory, Park Road, Islamabad, Pakistan

**Keywords:** Attenuated virus, Foot-and-mouth disease, In-activated virus, Recombinant virus, Transgenic vaccines

## Abstract

Control and prevention of foot and mouth disease (FMD) by vaccination remains unsatisfactory in endemic countries. Indeed, consistent and new FMD epidemics in previously disease-free countries have precipitated the need for a worldwide control strategy. Outbreaks in vaccinated animals require that a new and safe vaccine be developed against foot and mouth virus (FMDV). FMDV can be eradicated worldwide based on previous scientific information about its spread using existing and modern control strategies.

## Introduction

Foot-and-mouth disease (FMD) is one of the most economically and socially devastating diseases affecting cloven-hoofed livestock worldwide. It is caused by a highly variable RNA virus with seven serotypes (A, O, C, Asia 1, SAT 1, SAT 2, and SAT 3) and a large number of topotypes [1]. Millions of animals are sacrificed every year worldwide under FMDV eradication programmes [2]. FMDV has continuously circulated ever since after the first outbreak in America in 1870 [3,4]. Further, new subtypes of FMDV are continuously evolving due to an infinite mutation rate in the RNA genome of the virus [5].

Over the last few decades, disease-free countries have primarily adopted the strategy of slaughtering carrier animals combined with transport restrictions and other sanitary measures. Additionally, rapid vaccination is applied to limit the spread of infection in outbreak regions [6,7].

Currently, inactivated vaccines are used as a major tool in FMD eradication programmes in Europe as well as other parts of the world. However, these vaccines have a number of limitations such as propagation of virulent virus, threat of virus escape from manufacturing sites, limited shelf-life, and booster injection requirement after 4–12 months [8]. Sterility, safety, cost-effectiveness, easy delivery, and long-lasting immunity against multiple serotypes are additional challenges associated with conventional inactivated vaccines [8].

Recently, transgenic vaccines were demonstrated as a novel and safe strategy for the control and prevention of FMD. Specifically, animal fodder-based edible transgenic vaccines containing protein-expressing viral genomes are feasible to immunize animals. Many studies have suggested that FMD plant-derived edible vaccines will become common within the next few years [9].

## Review

### Continent disease spread

Control of FMD is difficult due to variations in viral serotype and consistency, effectiveness of control measures, and emergence of new subtypes. FMD outbreaks also originate from transportation of carrier animals to susceptible populations or disease-free regions. Moreover, prevalence of FMD increases due to seasonal or periodic cycling, host susceptibility, and predisposal to epizootic risk [10]. There are still many gaps in our understanding of FMD, especially in Asian countries. Rapid investigation of outbreaks samples and interpretation of data are made possible due to recent development of tools and techniques. Independent and collaborative works by various national animal health services, key control initiatives, laboratory networks, and many other groups have improved our knowledge of FMD [10].

Many countries have obtained FMD-free status from the International Organization of Animal Health (OIE) with or without vaccination. However, FMD has reemerged in previously disease-free countries due to increased viral escape from vaccinated animals and import of animal products from FMDV-circulating countries. Many countries have maintained their disease-free status by strict monitoring and culling of infected animals [11,12]. The southern part of South America achieved FMD-free status from the OIE in the late 1990s with the help of a strict eradication program, and other countries such as Uruguay, Argentina, Paraguay, and Brazil achieved FMD-free status via vaccination in 1994, 1997, 1997, and 1998, respectively [13].

Recently, FMD has reemerged in Japan and South Korea. Japan first achieved FMD-free status without vaccination in 2000, and FMD O serotype has not been reported in Mongolia and Russia since 2003 and 2004, respectively. In 2010, FMD serotype A and O outbreaks were reported in South Korea and Japan, respectively. However, FMD A outbreak in South Korea was controlled by March 2010 while the FMD O outbreak in Japan was controlled by June 2010 [12]. FMD outbreaks of serotype O continue to pose a threat to livestock industries in this region (Table 1) [14].

**Table 1:** **Countries with FMD free status in 2011 according to OIE [** 12 **]**

Albania	Germany	New Caledonia
Australia	**Greece**	**New Zealand**
Austria	**Guatemala**	**Nicaragua**
Belarus	**Guyana**	**Norway**
Belgium	**Haiti**	**Panama**
Belize	**Honduras**	**Poland**
Bosnia and Herzegovina	**Hungary**	**Portugal**
Brunei	**Iceland**	**Romania**
Canada	**Indonesia**	**San Marino**
Chile	**Ireland**	**Serbia**
Costa Rica	**Italy**	**Singapore**
Croatia	**Japan**	**Slovakia**
Cuba	**Latvia**	**Slovenia**
Cyprus	**Lesotho**	**Spain**
Czech Rep.	**Lithuania**	**Swaziland**
Denmark	**Luxembourg**	**Sweden**
Dominican Republic	**Madagascar**	**Switzerland**
El Salvador	**Malta**	**Ukraine**
Estonia	**Mauritius**	**United Kingdom**
Finland	**Mexico**	**United States of America**
Former Yug. Rep. of Macedonia	**Montenegro**	**Vanuatu**
France	**Netherlands**	

### Major causes of FMD spread in Asian/developing countries

Asian countries suffering from FMD outbreaks often lack coordinated or serious mandatory measures for control of this disease. Further, movement and exchange of animals and animal products across neighboring countries are very common. The amount of FMD vaccines produced locally is insufficient to fulfill the demands of large populations of animals in developing countries [5,15,16]. Moreover, FMD outbreaks among vaccinated animals in this region may be due to poor vaccine quality, lack of knowledge of circulating subtypes, and suboptimal vaccination strategies such as single vaccine injection without any booster [15].

### Virus distribution

Serotypes O, A, and Asia 1 are continuously circulating in many FMD endemic countries in Asia, Europe, and as well as Africa. Moreover, serotype C was reported in the Philippines in 1995, whereas SAT 1, 2, and 3 are common in African countries. Recently, disease-reporting transparency has improved due to increased field surveillance, outbreak investigation, and submission of virus samples for analysis by central reference laboratories such as the World Reference Laboratory (WRL) and FAO/OIE Reference Laboratory. However, efforts are still insufficient for comprehensive control and complete disease eradication [10,17].

#### Serotype O

FMDV serotype O is the predominant serotype of FMD worldwide. It is the most prevalent serotype in many parts of Africa, the Middle East such as Pakistan, and some parts of Europe. However, an accurate genetic explanation for the higher prevalence of O serotype is not yet available [18,19]. Serotype O has been responsible for severe disease outbreaks in Taiwan, Korea, Pakistan, Iran, Afghanistan, Israel, China, North Korea, and Bulgaria [17,20,21]. O1Manisa vaccine has been proven to be a robust immune dominant strain in many FMD O outbreaks, but it still does not protect against all epidemics. Moreover, several other O serotype vaccines have been used to improve vaccine efficacy for circulating outbreaks [10].

#### Serotype A

Members of this serotype show high antigenic diversity and no cross-protection between strains [16,22-24]. Genetic recombination is more common in serotype A than in other serotypes of FMD [25,26]. Serotype A is prevalent in ruminant populations of Thailand and Malaysia. The most recent outbreaks of serotype A were reported in Pakistan, Turkey, Egypt, India, China, and South Korea. Serotype A has been successfully controlled and eradicated in South Korea [17]. Different vaccines for serotypes A confer variable levels of protection. A Iran-05, A22 Iraq, and A24Cruzeiro serotypes were found to be very useful as vaccines against serotype A [10,17].

#### Serotype Asia 1

Serotype Asia 1 is the most antigenically stable serotype and shows relatively low levels of antigenic variation, although it is still capable of antigenic drift. Various historical epizootics have been reported mainly in Southeast Asian countries. Recently, serotype Asia 1 was shown to affect ruminants in China, Pakistan, Bahrain, Iran, Turkey, and Afghanistan in a cyclical pattern [17]. The Asia 1/Shamir immune dominant serotype has been proven to be a very valuable serotype to control outbreaks in Asian countries and is still recommended to address FMD Asia 1 outbreaks [17].

#### Serotypes SAT 1, 2, 3

SAT (Southern African Territories) serotypes are usually found in Africa, but a few outbreaks were recorded in Saudi Arabia and Kuwait in 2000 [27]. SAT have genetically more diverse FMD serotypes than other regions [28]. SAT 1 was reported in 2003, 2006, and 2009 in South Africa, whereas SAT 2 as reported in South Africa, Botswana, and Tanzania in 2008, 2010, and 2012 respectively. SAT 3 was last reported in 2006 in South Africa [17].

#### Serotype C

Sporadic outbreaks of serotype C have been reported in South America, East Africa, and Pakistan between 2000 and 2006 [29]. There have been no major epidemics in the last 20 years of this serotype [29]. Use of serotype C in vaccination may actually increase the risk of vaccine-induced outbreaks [30].

### FMDV genome and its role in infection

FMDV is an infectious RNA virus divided into three major functional regions. It comprises a 5′ non-coding regulatory region, protein-coding (L/P1, P2, and P3) region, and 3′ non-coding regulatory region. The protein P1 coding region encodes four structural capsid proteins, whereas P2–P3 regions encode non-structural proteins for replication and viral maturation. The functions of the non-structural proteins are still poorly understood [6,31].

#### Initiation of infection

FMDV infection is initiated by attachment of the RGD loop of viral capsid protein (VP1) to host surface integrins on target cells. This interaction between virion and cells is altered in some cell cultures in which a selected stretch of VP3 binds to heparin-like moieties on the cell culture surface [32]. AVB3 (Alpha V Beta), AVB5, and AVB6 integrins are virus attachment receptors in cattle [33].

#### Virus immune response and vaccines

Insufficient FMDV immunity can be attributed to the epitope between amino acids 140 to 160 having affinity for only B lymphocytes and not T lymphocytes. Identification of T lymphocyte-stimulating epitopes is thus a requirement for future vaccines. The three dimensional structure of FMDV includes a G-H loop in VP1 [4]. This G-H loop (highly conserved arginine-glycine-aspartic acid sequence) participates in binding to cell receptors [6,34,35]. Moreover, viruses containing a single point mutation in the RGD segment of VP1 regain virulence upon restoration of the RGD sequence [36]. However, RGD-deleted vaccines perform similar/better than BEI-inactivated ones with respect to protection against challenge and induction of immune response. Thus, an effective vaccine can be prepared by deletion of cell-binding sites from the virus using a genetic engineering approach [37].

### Current major vaccines

Vaccination is a major approach for controlling the spread of FMD. Inadequate safety and disease protection associated with conventional (inactivated or attenuated) vaccines has precipitated the need to develop effective and safe FMD vaccines. Adequate epidemiological data and revaccination times for different circulating serotypes are important for control of FMD in endemic regions [38]. Production of FMD vaccine requires large-scale antigen propagation, viral treatment for loss of pathogenicity, and adjuvant addition to enhance the immune response [39]. Good quality vaccines will allow avoidance of production loss and incidence of FMD [40]. Oil adjuvant of the Montanide series appears to be a promising candidate for a new generation of FMD vaccines [41]. Previously developed FMD vaccines were mostly ineffective [8] (Tables 2 and 3).

**Table 2:** **FMD free areas without vaccination [** 12 **]**

Argentina	Moldova
Botswana	Namibia
Brazil	Peru
Colombia	Philippines
Malaysia

**Table 3:** **Priority of different serotype of foot and mouth disease for vaccination**

**High priority serotypes**	**Medium priority serotypes**	**Low priority serotypes**
O Manisa	A Eritrea	A15 Bangkok related strain
O BFS or Campos	A Iran ’96	A87 Argentina related strain
A24 Cruzeiro	SAT 2 Zimbabwe	C Noville
Asia 1 Shamir	A Iran 87 or A Saudi Arabia 23/86	SAT 2 Kenya
A Iran-05	A Malaysia 97	SAT 1 Kenya
A22 Iraq	A Argentina 2001	SAT 3 Zimbabwe
SAT 2	O Taiwan 97	A Kenya
-	A Iran	-

#### Inactivated and attenuated vaccines

Inactivated vaccines are commonly used and effective tools to address FMDV outbreaks, but their production is expensive and is associated with risk of disease spread [38]. Virus propagated on cell culture (BHK-21) and chemically inactivated by binary ethyleneimine has been shown to be an effective vaccination protocol. Vaccine inactivated by aziridine and acetylethyleneimine and mixed with adjuvant such as aluminum hydroxide or saponin has been used at large scale due to its reliability and effectiveness [34]. In the early 1990s, studies revealed that vaccinated animals lack an immune response to non-structural proteins (NSPs) from viruses. This information can be used for serological screening of infected and carrier animals during vaccination. The manufacturers always face the problem of incomplete inactivation of virus along with screening of vaccinated and non-vaccinated animals [36,42].

Moreover, vaccine production against individual FMD serotypes is very challenging using a virus inactivation approach. Such vaccines may also lose potency and effectiveness from production to application due to errors in the cold delivery chain and field-limiting serotypes or topotypes. Further, short-lived immunity requires a booster, and inability to eradicate virus from carrier animals presents some limitations related to inactivated vaccines [34,43].

Recently, inactivated FMDV (iFMDV) vaccines (Cliptox-TM) have been shown to produce a specific antibody response in mucosal tissues and sera along with a Th1/Th2 response [44]. Previously, FMDV receptor or receptor-binding site-deleted/replaced FMDV attenuated vaccine has been explored for FMD protection in cattle [44,45]. Live attenuated vaccines prepared from leader proteinase-deficient serotype A12 and capsid containing 3C proteinase coding regions of Asia I/HNK/CHA/05 provide effective protection to cattle from FMDV. These studies validate the successful use of live/attenuated vaccine for FMD protection in endemic areas [46-48].

#### Peptide vaccine

Synthetic peptides are another promising technology for the control of FMD. A single epitope such as the G-H loop in the viral capsid and C-terminal region of VP1 correspond to B cell epitopes and stimulate an immune response with limited disease protection [49,50]. In addition, B cell affinity sites on VP1 as well as TH sites outside of VP1 are needed for production of neutralizing antibodies with high affinity. A peptide vaccine with both immunogenicity and antigenic cross-reactivity among serotypes was successfully developed from the entire G-H loop domain, flanking sequences (129–169), and artificial TH site of FMDV serotype O [31,51]. Immunization with peptides containing G-H loop either alone or in combination with an independent T cell epitope has been shown to induce 23% to 39% partial protection from viral infection [31,36].

Partial or complete removal of the VP1 G-H loop is a novel approach to develop FMDV negatively marked vaccines [52]. Dendrimeric peptide vaccine specifically induces high titers of FMDV-neutralizing antibodies and activates an FMDV-specific T cell response in pigs. Animals immunized with peptide vaccine are protected against specific FMDV challenge [53]. Peptide vaccines have limitations such as incomplete protection due to a limited number of antigenic sites that interact with the immune system, discontinuous antigenic sites on VP1, and the quasi species nature of the virus [51]. These limitations of peptide vaccines allow for different FMDV antigenic variants, resulting in outbreaks in vaccinated animals. Future peptide vaccines should have advanced and paramount viral structures to induce effective and complete immune responses [54].

#### Recombinant protein of FMD

Recombinant proteins are an alternative immunization method and are based on a set of effective epitopes within a single polypeptide chain. B and T cell polyepitope proteins can also be used to induce an effective immune response [55]. In previous studies, FMD synthetic polypeptides were shown to protect laboratory animals such as mice, rabbits, and guinea pigs. However, there is still no recombinant FMD vaccine commercially available for farm animals [56,57]. It has previously been shown that empty FMDV capsids are capable of eliciting the same antibody response as infectious FMDV particles [58]. Recombinant vaccine from B cell epitopes of VP1 and VP4 as well as T cell epitopes of proteins 2C and 3D was previously developed in *E.coli* or *N. benthamiana* plants using a phytoviral expression system. Immunization of guinea pigs with purified proteins has been shown to induce an efficient immune response against FMDV serotype O/Taiwan/99 as well as protection against homologous viral challenge [59]. Therefore, recombinant polyepitope viral proteins may be used as commercially available vaccination tools for the control and prevention of FMD in the future.

#### FMDV live vector vaccine

A promising novel FMD vaccination approach was developed using replication-defective human adenovirus serotype 5 (Ad5) containing FMDV transgenes [60]. Adenovirus-vectored vaccines with interferon or FMDV capsid proteins co-expressed with viral protease for processing have been shown to confer protection against FMD in pigs and cattle [61]. Previously, an experimental vaccine was also developed using replication-defective human adenovirus serotype 5 (Ad5) containing transgenes from FMDV P1 (coding for capsid proteins), NSP 2A, 2B, nearly all 3B, and 3C protease [62]. In another study, Ad5-A24-modified candidate also successfully protected animals against challenge with homologous FMDV. Similarly, FMD construct without 3D (polymerase) NSP portion was used to successfully differentiate among FMDV infected and vaccinated animals [63].

Pseudorabies virus-derived virus-like particles (VLPs) are highly immunogenic and helpful for safe production of FMD proteins in vaccinated animals [48]. Previously, single vaccination with an empty capsid from FMDV serotype Asia I/HNK/CHA/05 expressed by a silkworm baculovirus expression vector protected 80% of cattle from virulent homologous virus [64]. In the future, adenovirus or other viral-associated recombinant vaccines may be successful commercial candidates for the control and prevention of FMD.

#### Transgenic vaccine in plants

Transgenic vaccines in plants were first discussed by Mason [65]. Such vaccines are now considered as a promising option for linear epitopes. Further, FMDV edible vaccines in transgenic plants as bioreactors may overcome problems with cold storage and transportation of inactivated vaccines [6,9,66,67]. Previously, VP1 structural proteins were successfully expressed in Arabidopsis thaliana, alfalfa, and potato plants [66,68]. Lower expression and detection of transgenic proteins in plants are two major limitations to the application of edible vaccines. Reporter β glucuronidase gene was shown to facilitate rapid screening and identification of a number of transgenic plants. The selected plants developed strong and protective antibody responses against virulent FMDV in experimental hosts [9]. FMDV VP1 protein has been expressed in transgenic plants, and successful immunization in mice was also reported from China and Argentina [69,70].

Many studies have been conducted to develop FMD edible vaccines with an effective immune response in plants such as tomato, Arabidopsis thaliana, potato, Chlamydomonas chloroplasts, and tobacco [59,65,71-75]. Tissue-specific promoters increase FMD transgene expression at specific locations such as seeds for a specific time period in edible vaccines [1,65]. In a previous study, a novel oral immunization system was successfully developed against FMD using structural proteins (VP1) from serotypes O and Asia 1 in maize, and both transgenes were stably transmitted to the next generation [54]. Recombinant vaccines in transgenic plants such as cereals lack complications related to viral or prion adulteration in vaccines, along with many other benefits. Production of recombinant vaccines in plants is very economical and reduces transportation and storage costs [76]. Moreover, direct oral administration with multiple components makes edible vaccines more valuable [77]. Antigen expression in transgenic plants is more useful for experimental and commercial animal vaccine development as compared to classical methodologies.

## Conclusion

Control and eradication of FMD from endemic regions are only possible by combined efforts of the international community to produce cost-effective and environmentally friendly vaccines against circulating FMDV serotypes. Vaccination can be an effective control measure depending on local epidemiological and scientific disease information. The effectiveness of heterologous vaccination should also be studied with respect to antigenic matching of circulating serotypes with immediate selection of effective vaccines during outbreaks. FMD vaccination strategies, vaccine production, storage, and transportation are real practical challenges, especially in developing countries. In this scenario, transgenic vaccines in plants are attractive alternatives to conventional FMD vaccines. Plants can be grown efficiently at large-scale and easily delivered. However, production of FMD chimeric plant-based vaccines from local isolates is a real challenge worldwide. Sequence analysis of circulating virus is also very important for continuous assessment of mutation and antigenic changes in the viral genome. The vaccine should be improved with circulating serotype in case of any genetic difference from the field isolate. Disease-free countries must pay special attention to protect their livestock from FMD-infected animals and animal products. Modern transgenic vaccination can be used to lower the risk of disease in FMD-free countries and can help these countries to maintain their disease-free status.

Institutional review board of Institute of Molecular Biology and Biotechnology, Bahauddin Zakariya University, approved this study.
